# The role of monovalent cations in the ATPase reaction of DNA gyrase

**DOI:** 10.1107/S1399004715002916

**Published:** 2015-03-27

**Authors:** Stephen James Hearnshaw, Terence Tsz-Hong Chung, Clare Elizabeth Mary Stevenson, Anthony Maxwell, David Mark Lawson

**Affiliations:** a Department of Biological Chemistry, John Innes Centre, Norwich Research Park, Norwich NR4 7UH, England

**Keywords:** DNA gyrase, ATPase domain, ATPase activity, GHKL superfamily, monovalent cations

## Abstract

Four new crystal structures of the ATPase domain of the GyrB subunit of *Escherichia coli* DNA gyrase have been determined. One of these, solved in the presence of K^+^, is the highest resolution structure reported so far for this domain and, in conjunction with the three other structures, reveals new insights into the function of this domain. Evidence is provided for the existence of two monovalent cation-binding sites: site 1, which preferentially binds a K^+^ ion that interacts directly with the α-phosphate of ATP, and site 2, which preferentially binds an Na^+^ ion and the functional significance of which is not clear. The crystallographic data are corroborated by ATPase data, and the structures are compared with those of homologues to investigate the broader conservation of these sites.

## Abstract 2

New structures of the N-terminal 43 kDa fragment of the *E. coli* DNA gyrase B subunit reveal two discrete monovalent cation-binding sites that could have functional roles.

## 1. Introduction   

DNA topoisomerases are essential enzymes that are found across all kingdoms of life and, as a consequence, they represent valuable drug targets for both antimicrobial and anticancer therapies (Wang, 2002 ▶; Corbett & Berger, 2004 ▶; Chen *et al.*, 2013 ▶; Collin *et al.*, 2011 ▶). Type II topoisomerases, and DNA gyrase in particular, have been intensively studied; their role is to regulate DNA topology by generating a transient double-stranded break in one DNA duplex (the ‘G’ or gate segment) and passing another duplex (the ‘T’ or transported segment) through the break before resealing it. DNA gyrase functions as an A_2_B_2_ heterotetramer; each subunit is composed of several domains with distinct functions, and structural information is available for all of these domains. The focus of this study is the ATPase domain of *Escherichia coli* DNA gyrase, located in the B subunit, which functions as an ATP-operated clamp that captures the T segment prior to its passage through the G segment.

Several compounds, including the aminocoumarins and cyclothialidines, specifically target the ATPase site and, in general, function as competitive inhibitors (Maxwell & Lawson, 2003 ▶). Structures have been determined of a number of enzyme–inhibitor complexes and these show that the ligand-binding sites overlap with the nucleotide-binding site, thereby providing a molecular-level explanation of their inhibitory properties (Lamour *et al.*, 2002 ▶; Lewis *et al.*, 1996 ▶). X-ray structures have also helped to resolve the details of the catalytic mechanism whereby Mg-ATP is hydrolysed *via* a water-mediated nucleophilic attack (Jackson & Maxwell, 1993 ▶).

Looking further afield, structurally related ATPase domains are found in a number of other disparate enzymes, which are collectively referred to as the GHKL superfamily because they include DNA **G**yrase, **H**sp90, histidine **k**inases and Mut**L** (Dutta & Inouye, 2000 ▶). In addition to Mg-ATP, the binding of monovalent cations has also been reported adjacent to the nucleotide in a handful of GHKL superfamily enzymes, including DNA gyrase (although none are present in any deposited gyrase structure), prompting the suggestion that this finding might extend to other members of the superfamily (Hu *et al.*, 2003 ▶). Indeed, K^+^ is indispensable for nucleotide binding and hydrolysis in BCK (Machius *et al.*, 2001 ▶), but both Na^+^ and K^+^ support the ATPase activity of MutL (Hu *et al.*, 2003 ▶). Moreover, these findings have been linked to the observation that potassium ions stimulate ATP-dependent DNA supercoiling in *Bacillus subtilis* DNA gyrase and the hypothesis that they are required for closure of the ATP-operated clamp (Gubaev & Klostermeier, 2012 ▶). As a result, it has been suggested that accounting for these monovalent cations in the design of nucleotide analogues that target the ATPase sites of GHKL enzymes could help to minimize undesirable off-target effects (Hu *et al.*, 2003 ▶). Given the potential importance of monovalent cations in GHKL enzyme activity, we were prompted to investigate this further through X-ray crystallo­graphic and biochemical studies on the *E. coli* DNA gyrase ATPase domain.

## 2. Materials and methods   

### 2.1. Sample preparation   

The N-terminal 43 kDa fragment (exact molecular weight 43 254.8 Da) of *E. coli* DNA gyrase subunit B (GyrB43), comprising residues 2–393 of the 804-amino-acid wild-type sequence (UniProtKB/Swiss-Prot entry P0AES6), was produced using a modification of a previously published protocol (Jackson *et al.*, 1991 ▶). Expression plasmid pAJ1 was transformed into *E. coli* strain BL21 (DE3) pLysS and a 5 ml overnight culture was used to inoculate 1 l Luria–Bertani medium containing 100 mg ampicillin and 30 mg chloramphenicol. The cells were grown at 310 K to an OD_600_ of 0.4–0.6. Protein expression was induced by the addition of isopropyl β-d-1-thiogalactopyranoside to a final concentration of 0.5 m*M* and the culture was left shaking overnight at 298 K. The harvested cells were resuspended in TGED [50 m*M* Tris–HCl pH 7.5, 10%(*v*/*v*) glycerol, 1 m*M* EDTA, 1 m*M* DTT] and then lysed by freeze–thawing with liquid nitrogen. The cell debris was removed by centrifugation at 84 000*g* for 60 min at 277 K. The sample was subsequently purified using an ÄKTA FPLC system (GE Healthcare) using a two-column procedure. The sample was maintained at 277 K throughout and fractions that contained GyrB43 were identified using SDS–PAGE. The cell lysate was passed through a Q Sepharose column pre-equilibrated with TGED, which was then washed with excess TGED. The remaining bound protein was eluted with a 0.0–1.0 *M* NaCl gradient in TGED. Solid ammonium sulfate was added to the protein fraction containing the GyrB43 to a final concentration of 1.5 *M*. This was then loaded onto a 1 ml Phenyl Sepharose column pre-equilibrated with three column volumes of 1.0 *M* ammonium sulfate in TGED. The column was then washed with one column volume of 1.5 *M* ammonium sulfate in TGED. Any remaining bound protein was subsequently eluted by applying a 1.0–0.0 *M* ammonium sulfate gradient in TGED buffer. Eluted fractions containing GyrB43 were dialysed overnight against excess TGED containing 30%(*v*/*v*) glycerol. The dialysed protein was then aliquoted, flash-cooled in liquid nitrogen and stored at 193 K.

Prior to crystallization, the protein was buffer-exchanged into TGED containing the nonhydrolysable ATP analogue 5′-adenylyl-β,γ-imidodiphosphate (ADPNP) at a concentration of 40 µ*M* and then concentrated to ∼10 mg ml^−1^ using an Amicon Ultra centrifugal filter unit with a 10 kDa molecular-weight cutoff.

### 2.2. Crystallization and X-ray data collection   

All crystallizations were performed at a constant temperature of 291 K in 24-well hanging-drop vapour-diffusion format using VDX plates (Hampton Research) with a reservoir volume of 1 ml and drops consisting of 0.6 µl protein solution and 0.6 µl precipitant. Crystallization conditions were optimized from those reported previously (Jackson *et al.*, 1991 ▶) such that single crystals with dimensions of up to 200 × 100 × 50 µm grew within 24 h from 15–25%(*v*/*v*) PEG 3350, 2 m*M* MgCl_2_, 100 m*M* Tris–HCl pH 8.0. In subsequent experiments, crystals were grown from the same conditions with the addition of either 100 m*M* KCl or 100 m*M* NaCl or both 100 m*M* KCl and 100 m*M* NaCl. Prior to mounting, the crystals were cryoprotected in the corresponding precipitant solution supplemented with 10%(*v*/*v*) glycerol and then flash-cooled in liquid nitrogen using LithoLoops (Molecular Dimensions). The mounted crystals were stored in Unipuck cassettes (MiTeGen) prior to transport to the synchrotron. Crystals were subsequently transferred robotically to the goniostat on one of three beamlines at the Diamond Light Source (Oxfordshire, England; see Table 1 ▶) and maintained at 100 K with a Cryojet cryocooler (Oxford Instruments).

### 2.3. X-ray data processing and analysis   

All X-ray data were processed using the *xia*2 expert system (Winter, 2010 ▶) and the resultant data-collection statistics are summarized in Table 1 ▶. The crystals belonged to the ortho­rhombic space group *C*222_1_, with approximate unit-cell parameters *a* = 88, *b* = 141, *c* = 80 Å, α = β = γ = 90°, which are similar to those reported previously (Jackson *et al.*, 1991 ▶; Wigley *et al.*, 1991 ▶; Stanger *et al.*, 2014 ▶). The highest resolution data set, collected to 1.75 Å resolution from a crystal grown in 100 m*M* KCl (hereafter referred to as ‘K-only’), was used to solve and refine the first structure. One monomer (together with its associated ADPNP and magnesium ion) was taken from the deposited structure of *E. coli* GyrB43 (PDB entry 1ei1; Brino *et al.*, 2000 ▶) and used as the search template in *MOLREP* (Vagin & Teplyakov, 2010 ▶), which correctly placed one copy of this in the asymmetric unit to give a recognisable GyrB43 homodimer after application of the appropriate twofold crystallographic symmetry operator. Prior to rebuilding and refinement, the protein component was subjected to simulated-annealing refinement from a starting temperature of 5000 K using *PHENIX* (Adams *et al.*, 2010 ▶). The resultant model was completed through several iterations of refinement with *REFMAC*5 (Murshudov *et al.*, 2011 ▶) and interactive model building with *Coot* (Emsley & Cowtan, 2004 ▶). For refinement of the ADPNP ligand (three-letter code ANP), a CIF geometrical restraints dictionary was prepared using the *GRADE* web server (http://grade.globalphasing.org/cgi-bin/grade/server.cgi). Initially, solvent molecules were introduced automatically using *ARP*/*wARP* (Lamzin & Wilson, 1993 ▶), and these were then modelled and evaluated manually. Where appropriate, solvent molecules were replaced with metal ions, based on the nature, proximity and disposition of neighbouring atoms, as well as the temperature factor (when refined as water molecules) relative to those of these neighbouring atoms. It is important to note that by default *REFMAC*5 does not restrain the lengths of inter­actions involving waters molecules or ions, so these distances should not be biased by the atom type chosen for refinement. In the final stages, TLS refinement was used in *REFMAC*5 with a total of four TLS domains, which were defined using the *TLS Motion Determination* (*TLSMD*) server (http://skuld.bmsc.washington.edu/~tlsmd/; Painter & Merritt, 2006 ▶). The protein component of the final K-only model was used as a starting point for the modelling and refinement of the remaining structures reported here, which were all isomorphous. In each case, these coordinates were subjected to simulated-annealing refinement against the new data set using *PHENIX*. Thereafter, essentially the same refinement and modelling procedures were employed as above to complete these structures. For each, the same subset of reflections as that used for the K-only data set was omitted from the refinement procedure and used in the *R*_free_ calculation. The statistics of the final models are summarized in Table 2 ▶.

Two data sets were collected at longer wavelength (1.91 Å) to further probe the identities of metal ions through the use of anomalous difference Fourier maps. These were produced using phases calculated from the K-only model after the removal of all metal ions, simulated annealing as described above and re-refinement against the K-only data set. The resultant maps were inspected for significant positive peaks of electron density associated with atomic positions, which could then be assigned as anomalously scattering atoms. At a wavelength of 1.91 Å, the theoretical anomalous signals associated with the non-H atoms that could be present would be ranked as follows: K > Cl > S > P > Mg > Na >> O, N, C, where the theoretical Na signal would be roughly one eighth of the K signal (Supplementary Table S1).

All structural figures were prepared using *CCP*4*mg* (Mc­Nicholas *et al.*, 2011 ▶).

### 2.4. ATPase assays   

ATPase assays were carried out using a linked assay as described previously (Ali *et al.*, 1993 ▶) using an *E. coli* Gyrase ATPase Kit supplied by Inspiralis Ltd after dialysing the protein into TGED containing 50%(*w*/*v*) glycerol to remove the KCl present in the storage buffer. Briefly, assays were performed in microtitre plates with a reaction volume of 100 µl and a constant GyrB43 concentration of 20 µ*M*. The absorbance at 340 nm was measured continuously in a CLARIOstar plate reader (BMG LABTECH) and used to evaluate the oxidation of NADH (using an extinction coefficient of 6.22 m*M* ^−1^ cm^−1^), which relates stoichiometrically to the production of ADP.

## 3. Results   

### 3.1. Identification of monovalent cation-binding sites in the gyrase ATPase domain   

The N-terminal 43 kDa fragment of the *E. coli* DNA gyrase B subunit comprises the ATPase and transducer domains. The latter domain participates in nucleotide binding and plays a role in signalling the nucleotide-bound status to the rest of the heterodimer. In all the structures described here, GyrB43 adopts a so-called ‘restrained’ conformation that is characterized by a close association between the ATPase and transducer domains (Corbett & Berger, 2005 ▶).

**Figure 1:**
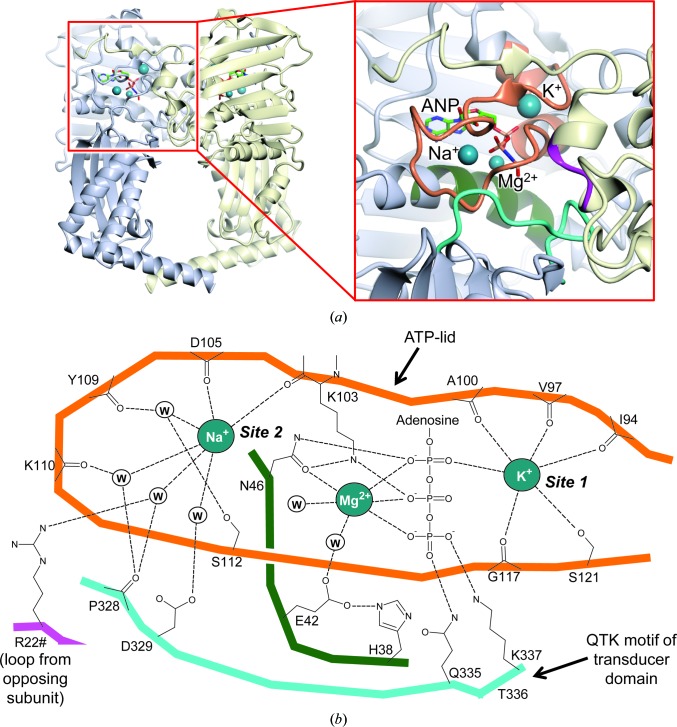
Overview of cation-binding sites in *E. coli* GyrB43. (*a*) Cartoon of the K-only model of the GyrB43 homodimer with an enlarged inset showing the relative positions of the metal-binding sites and the bound ADPNP (abbreviated to ANP). The slate grey and pale yellow colours define the two subunits, whilst the additional colours in the inset indicate the regions that participate in cation binding as shown in (*b*). These images are also reproduced as stereoviews in Supplementary Fig. S1. (*b*) Schematic showing details of sites 1 and 2 and the Mg-ATP binding site. The colours of the protein backbone correspond to the colours used in the inset in (*a*). Where possible the approximate spatial arrangement of the various structural elements has been preserved, with the exception of the motif bearing Arg22, which has been moved to the lower left-hand corner to aid clarity. H atoms have been omitted. Circles labelled ‘W’ indicate water molecules.

The K-only model was determined to 1.75 Å resolution, representing the highest resolution structure reported so far for GyrB43, or indeed for any equivalent topoisomerase fragment (Fig. 1 ▶ *a* and Supplementary Fig. S1*a*). As such, this offers the best prospect so far for the unambiguous identification of any metal ions bound to this protein fragment. Indeed, during refinement it became apparent that two sites occupied by water molecules could be convincingly remodelled as metal ions. In the first site, lying adjacent to the nucleotide-binding site at the base of a surface loop termed the ‘ATP-lid’ (Dutta & Inouye, 2000 ▶) and hereafter referred to as ‘site 1’, the ‘water’ molecule was octahedrally coordinated by six O atoms, four of these being provided by backbone carbonyls, one by a Ser side chain (Ser121) and one by the α-phosphate of ADPNP (Figs. 1 ▶ *b* and 2 ▶ *a*). Whilst the average interaction length of 2.8 Å was comparable to that expected for a hydrogen bond to a water molecule, the refined temperature factor was significantly lower than the average value for the interacting atoms, suggestive of a species that is significantly more electron-dense than a water molecule. Taken together, the evidence indicated that site 1 was occupied by a K^+^ ion. In support of this conclusion, subsequent refinement with a K^+^ ion in this position gave a temperature factor comparable to the average value of the interacting atoms (Supplementary Table S3). In the second site, located towards the tip of the ATP-lid and hereafter referred to as ‘site 2’, the ‘water’ molecule was also octahedrally coordinated by six O atoms, but in this case only two of these were provided by backbone carbonyls and the remaining four were provided by other water molecules (Figs. 1 ▶ *b* and 3 ▶). This time the refined temperature factor was comparable to the average value of the interacting atoms (suggestive of a species with comparable electron density to a water molecule), but the average interaction length of 2.5 Å was at the lower end of that expected for a hydrogen bond to a water molecule. Taken together, the evidence indicated that site 2 was occupied by an Na^+^ ion. In support of this conclusion, subsequent refinement with an Na^+^ ion in this position gave a temperature factor comparable to the average value of the interacting atoms (Supplementary Table S3).

When the K-only model was superimposed on an anomalous difference Fourier map calculated using data collected from a similarly prepared crystal but at a longer wavelength of 1.91 Å (K-anom data), the highest peak in the map corresponded to the species present in site 1, consistent with it being a K^+^ ion (Supplementary Fig. S2*a*). Overall the map was quite noisy, but of the top 11 peaks two were associated with ADPNP phosphates and four were associated with Met or Cys S atoms; peak 10 corresponded to a ‘water’ molecule and peak 11 corresponded to the species present in site 2 (Supplementary Fig. S2*c*). The height of the latter was ∼35% that of the site 1 peak and therefore was too significant to result from an Na^+^ ion. We concluded that this site was most likely partially occupied by a K^+^ ion. However, given that the refined geometrical parameters of the modelled ion were entirely consistent with Na^+^, a mixed-occupancy site was not modelled. The ‘water’ molecule corresponding to peak 10 had three amine groups as ligands at distances of 3.2–3.5 Å, strongly indicative of it most likely representing a chloride ion, which was subsequently modelled satisfactorily in this position. Indeed, a chloride ion was inserted here in the ensuing structures. As expected, there were no peaks associated with the Mg^2+^ ions, which would have a relatively low anomalous signal at this wavelength (Supplementary Table S1).

The completed K-only model, consisting of 384 amino-acid residues, 249 water molecules, one ADPNP molecule, one magnesium ion, one potassium ion, one sodium ion and one chloride ion, was refined at 1.75 Å resolution to *R*_work_ and *R*_free_ values of 0.179 and 0.204, respectively, and satisfactory geometrical parameters, with *MolProbity* (Chen *et al.*, 2010 ▶) reporting a single Ramachandran outlier, namely Asn178 (Table 2 ▶). Nevertheless, inspection in *Coot* showed that the latter was well resolved and unambiguously defined by the electron density.

**Figure 2:**
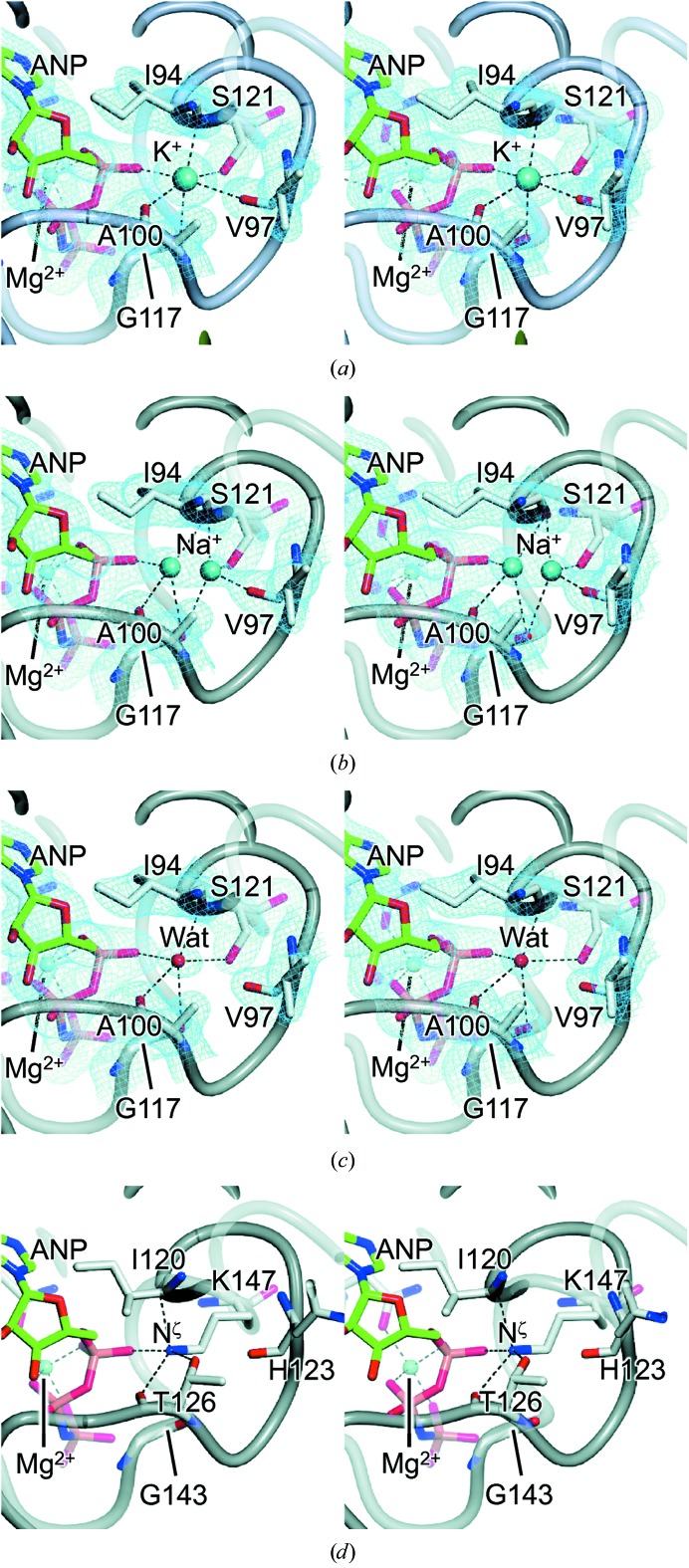
Stereoviews showing detail of monovalent cation-binding site 1 in the models of *E. coli* GyrB43 presented here. (*a*) K-only model refined with a fully occupied K^+^ ion at 1.75 Å resolution; the equivalent site in the K+Na model is virtually identical (not shown). (*b*) Na-only model refined with a discretely disordered Na^+^ ion at 1.90 Å resolution. (*c*) No-salt model refined with a fully occupied water molecule at 1.95 Å resolution (all electron-density maps shown are of the form 2*mF*_obs_ − *DF*_calc_ and are contoured at 1.0σ). (*d*) Equivalent site in yeast topoisomerase II (PDB entry 1pvg) showing how the N^ζ^ atom of Lys147 effectively substitutes for the K^+^ ion seen in the GyrB43 K-only structure.

The second structure was determined using a data set collected at 1.90 Å resolution from a crystal grown in 100 m*M* NaCl (‘Na-only’ data set). The resultant model was essentially identical to the K-only model at the nucleotide-binding site, site 2 and the chloride site. However, the electron density at site 1 was elongated and weaker than expected for a K^+^ ion. The latter could however be modelled satisfactorily as a disordered Na^+^ ion occupying two discrete locations, each with 50% occupancy, within the binding site (Fig. 2 ▶ *b*; Supplementary Table S4). Overall the structure was closely similar to the K-only structure, giving an r.m.s. deviation of only 0.16 Å after superposition (Supplementary Table S2).

Refinement against a data set collected at 1.92 Å resolution from a crystal grown in the presence of both 100 m*M* KCl and 100 m*M* NaCl (‘K+Na’ data set) gave a model that was essentially equivalent to the original K-only structure, *i.e.* containing a K^+^ ion in site 1 and an Na^+^ ion in site 2. After superposition, the two structures gave an r.m.s. deviation of only 0.13 Å (Supplementary Table S2). Moreover, the geometric parameters of the monovalent cation-binding sites were closely similar to those of the K-only model (Supplementary Tables S3 and S5). An anomalous difference Fourier map calculated using data collected from a similarly prepared crystal at a wavelength of 1.91 Å (K+Na-anom data) gave peaks corresponding to the site 1 species, the ADPNP phosphates (Supplementary Fig. S2*b*), several Met/Cys S atoms and the previously mentioned chloride ion. However, this time there was no peak associated with site 2 (not shown), which was indicative of this site being fully occupied with an Na^+^ ion.

The last structure was determined using a data set collected at 1.95 Å resolution from a crystal grown using a precipitant solution containing neither KCl nor NaCl (‘No-salt’ data set). This time the density at site 1 was relatively weak, less than would be expected for a K^+^ ion, and indicated an ordered species, in contrast to the elongated density that was attributed to a discretely disordered Na^+^ ion in the Na-only model. The distances to five of the six O atoms that form the surrounding octahedral coordination sphere lay in the range 2.75–3.22 Å, being entirely consistent with hydrogen bonds to a water molecule. The distance of 3.38 Å to the sixth potential ligand (Val97 O) was somewhat outside the normal range expected for hydrogen bonds and is therefore unlikely to contribute to binding the water molecule (Fig. 2 ▶ *c*). Refining the bound species as a water molecule gave a temperature factor comparable to the average for the surrounding liganding atoms. Despite the exclusion of NaCl from the crystallization and cryoprotection solutions, site 2 was again modelled satisfactorily with a fully occupied Na^+^ ion (Supplementary Table S6). Overall the structure was closely similar to the three structures described above, giving r.m.s. deviations of only 0.242, 0.225 and 0.188 Å after superposition for the K-only, Na-only and K+Na structures, respectively (Supplementary Table S2).

### 3.2. Comparison with structural homologues   

Interrogation of the Protein Data Bank through the *DALI* server (http://ekhidna.biocenter.helsinki.fi/dali_server/; Holm & Sander, 1995 ▶), using the K-only model as the template, retrieved many close structural homologues, with over 500 protein chain entries giving a *Z*-score exceeding 10.0. These were sifted for redundancy and relevance (*e.g.* structures without bound nucleotide were ignored), whilst at the same time screening for the presence of cations in locations corresponding to sites 1 and 2 of GyrB43. Within the remaining structures, the architecture of site 1 was generally well conserved and frequently contained a bound water molecule, although only in the structure of *E. coli* MutL (PDB entry 1nhi; Hu *et al.*, 2003 ▶) was a K^+^ ion modelled here (Table 3 ▶). However, we cannot rule out the possibility that K^+^ could be modelled into site 1 for other PDB entries, but this cannot be performed with confidence for the lower resolution structures. We did, however, notice in the ADPNP-bound structure of the 43 kDa N-terminal domain of *E. coli* ParE determined at 2.1 Å resolution (PDB enry 1s16; Bellon *et al.*, 2004 ▶) that the Mg^2+^ ion associated with the nucleotide in each monomer of the homodimer in fact occupied site 1 with identical ligands to *E. coli* GyrB43 (Table 3 ▶). Closer inspection showed that the refined cations had temperature factors of less than half of the average value for the liganding atoms. Moreover, the average interaction lengths were 2.8 Å. Therefore, we conclude that these Mg^2+^ ions should be remodelled as K^+^ ions. Curiously, the more usual Mg^2+^ site, where the cation is bound by all three phosphate groups of the nucleotide, was vacant.

For completeness, we also interrogated the PDB with amino-acid sequences of representative GHKL family members, looking for entries that had greater than 30% identity to the query sequences and containing K^+^ or Na^+^ ions in sites relevant to this study. The sequences used were *E. coli* GyrB (UniProtKB/Swiss-Prot entry P0AES6), yeast DNA topoisomerase II (UniProtKB/Swiss-Prot entry P06786), *E. coli* MutL (UniProtKB/Swiss-Prot entry Q3YUH3), *Caenorhabditis elegans* heat-shock protein 90 (HSP90; UniProtKB/Swiss-Prot entry Q18688), *Thermotoga maritima* chemotaxis protein CheA (UniProtKB/Swiss-Prot entry Q56310), human mismatch-repair endonuclease PMS2 (UniProtKB/Swiss-Prot entry P54278) and rat branched-chain α-ketoacid dehydrogenase kinase (BCK; UniProtKB/Swiss-Prot entry Q00972). Only the BCK search yielded new hits: a total of 12 structures with K^+^ in the equivalent of site 1, all of which were structures of rat BCK (Machius *et al.*, 2001 ▶; Tso *et al.*, 2013 ▶). Relative to the initial *DALI* search based on structural homology, these structures fell outside our cutoff, with a representative *Z*-score being 7.5 for PDB entry 1gkz (Machius *et al.*, 2001 ▶; Table 3 ▶).

Intriguingly, we noted that in the eukaryotic topoisomerase II (topo II) structures from yeast and human (PDB entries 1pvg and 1zxn, respectively; Classen *et al.*, 2003 ▶; Wei *et al.*, 2005 ▶) the residue corresponding to Ser121 of *E. coli* GyrB was substituted by a Lys. As a consequence, the site 1 cavity was occupied by a Lys side chain, such that the N^ζ^ atom overlapped with the K^+^ ion of our K-only model after superposition (Fig. 2 ▶ *d*; Table 3 ▶). Thus, eukaryotic topo II has a positive charge ‘hard-wired’ into the protein structure at this location.

DNA gyrase and topo II are both examples of type IIA topoisomerases. However, there is also structural information on the more distantly related type IIB topoisomerases, for example topoisomerase VIB from *Sulfolobus shibatae* (PDB entry 1mx0; Corbett & Berger, 2003 ▶). Despite the relatively low sequence identity (*e.g.* ∼16% *versus E. coli* gyrase), the GHKL domains superpose well and reveal that, like topo II, topoisomerase VIB also has a Lys side chain occupying the equivalent of the site 1 cavity adjacent to the bound nucleotide (Table 3 ▶).

By contrast to the situation in site 1, there was much more variability in the region corresponding to site 2, such that in most of the structural homologues no equivalent site was apparent and in none was a monovalent cation evident.

In the previously deposited *E. coli* GyrB43 structures, we also looked for conservation of the chloride site (Table 3 ▶). In the structure determined by Brino and coworkers (PDB entry 1ei1; Brino *et al.*, 2000 ▶), the equivalent site is occupied by a sulfate ion. Inspection of the three recently deposited *E. coli* GyrB43 structures (Stanger *et al.*, 2014 ▶) reveals that in two of these this site is empty, whilst in the fourth (PDB entry 4pu9) it is occupied by a water molecule. Analysis of *B* factors and interatomic distances suggests that this could also be modelled satisfactorily as a chloride ion.

### 3.3. The effect of K^+^ and Na^+^ on GyrB43 ATPase activity   

We evaluated the effect of different metal ions on the ATPase activity of GyrB43 by the addition of increasing concentrations of either KCl or NaCl (from 0.0 to 1.0 *M*) to the assay mixture. We then tested the metal-ion preference with a competition ATPase assay, keeping a constant concentration of 500 m*M* NaCl in each reaction mixture while increasing the KCl concentration.

We found that GyrB43 is capable of hydrolysing ATP in the presence of either Na^+^ or K^+^, but that the rates of ATP turnover were significantly higher when K^+^ was used. We also observed a preference for GyrB43 to utilize K^+^ over Na^+^ in the competition experiment, with the ATPase rates increasing above the maximum value recorded for Na^+^ alone when the K^+^ concentration exceeded 300 m*M* (which is still 200 m*M* lower than the constant Na^+^ concentration; Fig. 4 ▶). We equate these effects to the occupancy of site 1, since it lies adjacent to the site of ATP hydrolysis and is capable of binding both Na^+^ and K^+^ ions, but has a clear preference for binding a K^+^ ion even when presented with both K^+^ and Na^+^ ions. Thus, these results are consistent with our crystallographic data.

By contrast, site 2 is more remote from the ATPase site and our structural studies indicate that it favours Na^+^ ions even in the presence of excess K^+^ ions. We conclude that it not involved in the K^+^-stimulated increase in ATPase activity and is most likely to play no direct role in ATP hydrolysis.

## 4. Discussion   

Here, we have shown that there are two monovalent metal ion-binding sites in the ATPase domain of the DNA gyrase B protein. Site 1 corresponds to the monovalent metal-ion site reported previously (Hu *et al.*, 2003 ▶). It lies on the opposing side of the phosphate tail of the nucleotide to the magnesium ion and is inaccessible to bulk solvent (Fig. 1 ▶ and Supplementary Fig. S1). Given that the bound species interacts directly with the α-phosphate of ADPNP, it seems likely that the site is occupied concomitantly with the binding of Mg-ATP. In this study, we show that in the presence of KCl the site is preferentially occupied by K^+^ even when NaCl is present at the same concentration (Fig. 2 ▶ *a*). However, when only NaCl is present Na^+^ is bound, giving rise to elongated electron density that can be modelled as two discrete half-occupancy Na^+^ sites (Fig. 2 ▶ *b*). In the absence of either KCl or NaCl, the site can be occupied by a water molecule (Fig. 2 ▶ *c*). The respective binding of K^+^, Na^+^ and water in this site in the K-only (and the closely similar K+Na model), Na-only and No-salt models is consistent with the evidence from the atomic *B* factors in the refined models. In all cases, the final *B* factor of the bound species is within 15% of the average value for the six potential liganding atoms (Supplementary Tables S3–S6).

The deployment of four backbone carbonyl O atoms to coordinate the ligand will impart a certain amount of rigidity to the site 1 cavity and is likely to reduce the ligand promiscuity at this site. Nevertheless, our data indicate that this site is capable of binding K^+^, Na^+^ or water. It is notable that the binding of K^+^ results in a small contraction in the site relative to the situation with a bound water (Table 4 ▶) and may reflect the more electrostatic nature of the interactions of the protein with this cation. As a result, when K^+^ is bound the interaction lengths are close to the ideal value of 2.84 Å (2.81 Å on average in the K-only model) proposed for K^+^—O distances (Harding, 2002 ▶). However, the comparative rigidity of the site prevents an even greater contraction to accommodate an ordered Na^+^ with Na^+^—O distances close to the ideal value of 2.42 Å (Harding, 2002 ▶). Instead, when Na^+^ binds our Na-only model shows that it effectively oscillates between two discrete locations separated by 1.74 Å, such that in each position it only makes effective interactions with a subset of the six possible liganding O atoms and, on average, these are longer than the ideal Na^+^—O separation (Supplementary Table S4). As result, there is no drawing in of the liganding O atoms and the cavity volume is similar to that seen when water is bound. If, as an approximation, we assume that the binding pocket is spherical, with a radius derived from the average interatomic distances of diametrically opposed O atoms (Supplementary Fig. S3), this value is 1.38 Å in the K-only structure (Table 4 ▶), being close to the ionic radius of K^+^, *i.e.* 1.33 Å (Glusker, 1991 ▶). By contrast, a value of 1.58 Å was measured for the Na-only structure, being significantly larger than the ionic radius of Na^+^, *i.e.* 0.95 Å (Glusker, 1991 ▶). Taken together, these observations are indicative of site 1 having evolved to preferentially bind K^+^.

**Figure 3:**
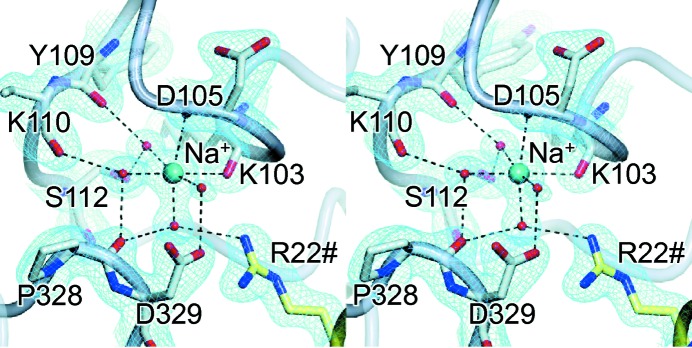
Stereoview showing detail of the monovalent cation-binding site 2 in the K-only model of *E. coli* GyrB43. The model was refined with a fully occupied Na^+^ ion at 1.75 Å resolution (the electron-density map is of the form 2*mF*_obs_ − *DF*_calc_ and is contoured at 1.0σ). Note that Arg22 is provided by the opposing subunit.

**Figure 4:**
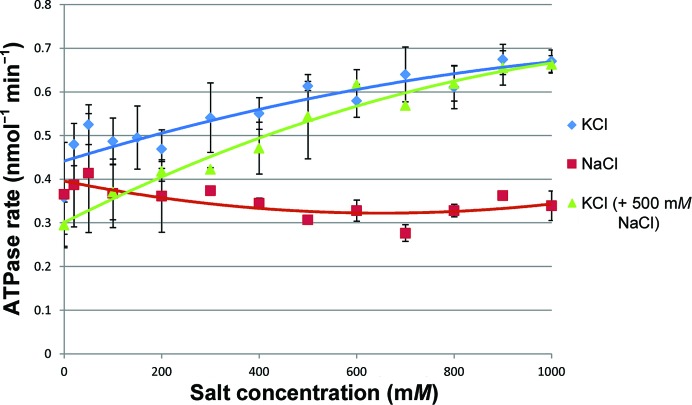
Variation in the ATPase activity of GyrB43 in response to changes in salt concentration. Data are shown for increasing NaCl and KCl concentrations, respectively, and for increasing KCl concentrations with a constant NaCl concentration of 500 m*M*. The rates shown are initial velocities; the data were fitted in *Microsoft Excel* using quadratic polynomial trendlines.

Since the occupant of site 2 makes direct contact with only two protein ligands, the remaining four interactions being with water molecules (Fig. 3 ▶), one would expect more flexibility here, resulting in a more relaxed ligand preference. Surprisingly, however, in all of the models presented in this study only Na^+^ refines satisfactorily in this site (Supplementary Tables S3–S5). Nevertheless, the appearance of a minor peak in an anomalous difference Fourier map calculated using the K-anom data set suggested a minor contamination with K^+^ in site 2 when this is the only monovalent cation present in the crystallization and cryoprotection solutions (Supplementary Fig. S2*c*). Initially, we were at a loss to explain why site 2 contained an Na^+^ ion in the K-only and No-salt structures, as this site would only be formed after the addition of ADPNP owing to the ATP-lid adopting a different conformation (or being disordered) in the absence of nucleotide. However, having looked back through all the reagents used, we realised that the EDTA component of the TGED buffer was in fact the disodium salt. Therefore, given that EDTA is at 1 m*M* in TGED, the protein would have been exposed to 2 m*M* Na^+^ ion when the ADPNP was added prior to the crystallization stage. Nevertheless, no Na^+^ ions were present in the cryoprotectant used for the K-only and No-salt data sets. We therefore propose that in these structures the species present in site 2 was a tightly bound Na^+^ ion that was not removed by the cryoprotection process.

**Figure 5:**
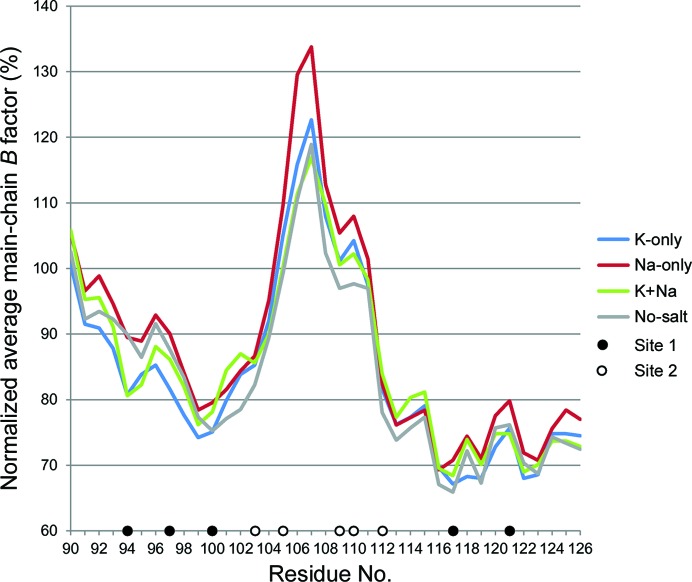
Plot of normalized average main-chain *B* factor for the ATP-lids of the four models of *E. coli* GyrB43 determined in this study. Main-chain *B* factors were averaged using *BAVERAGE* (Winn *et al.*, 2011 ▶), normalized by dividing by the overall average for all main-chain atoms and expressed as a percentage value.

The four structures presented here are closely similar, giving overall r.m.s. deviation values not exceeding 0.24 Å in pairwise comparisons of equivalent C^α^ positions and values not exceeding 0.18 Å in pairwise comparisons of the respective ATP-lids (residues 90–126; Supplementary Table S2). In the crystal, the ATP-lid is not constrained by crystal contacts, therefore if the species occupying site 1 has a direct influence on the stability of the domain, one might expect this to be reflected in the *B* factors of the ATP-lid. However, plots of averaged residue main-chain *B* factors normalized to the overall main-chain average were somewhat similar for the four structures (Fig. 5 ▶). Nevertheless, the values for the N-terminal portion of the ATP-lid, including residues 94, 97 and 100 from site 1, were noticeably lower for the K-only structure, and to a lesser extent for the K+Na structure, relative to the two other structures. Curiously, the values for the central portion encompassing site 2 were noticeably higher for the Na-only structure, despite site 2 being occupied by Na^+^ in all four models.

Previous work has shown that K^+^ ions stimulate ATP-dependent DNA supercoiling in *B. subtilis* DNA gyrase, and it was proposed that they are required for closure of the ATP-operated clamp (Gubaev & Klostermeier, 2012 ▶). The functional importance of K^+^ ions for other GHKL enzymes has also been demonstrated (Hu *et al.*, 2003 ▶; Shimomura *et al.*, 1988 ▶). Elsewhere, K^+^ ions have been shown to enhance the activity of other phosphotransferases, such as the molecular chaperone Hsc70, where K^+^ ions were also observed to be associated with the bound nucleotide in the crystal structure (Wilbanks & McKay, 1995 ▶). Our ATPase data are consistent with the current work, showing that K^+^ ions enhance the ATPase activity of GyrB43 when compared with an alternative metal-ion source (Na^+^) and that GyrB43 will preferentially utilize K^+^ ions over Na^+^ ions. Since our structural data reveal only a single K^+^-specific site in GyrB43, *i.e.* site 1, we propose that K^+^ ions stimulate ATPase activity through binding to this site. Given the proximity of the site to the nucleotide, it seems probable that K^+^ binding has a direct effect on catalysis rather than simply having a structural role. The triphosphate moiety of the nucleotide is bound almost exclusively by amide groups provided by both the protein backbone and the side chains of Asn46, Lys103, Gln335 and Lys337 in GyrB. Together with the Mg^2+^ ion, these protein ligands will enhance ATP hydrolysis through electrostatic stabilization of the transition state of the reaction. It seems likely that K^+^ also contributes to this effect. However, given its juxtaposition to the α-phosphate, rather than the γ-phosphate, its principal role may be to withdraw negative charge from the latter, thereby deshielding the P atom and increasing its susceptibility to the water-mediated nucleophilic attack that initiates hydrolysis (Jackson & Maxwell, 1993 ▶). We can only assume that the differential effects of K^+^ *versus* Na^+^ on catalytic activity are simply owing to their dissimilar ionic radii, with the dimensions of the site 1 cavity favouring the former cation. It is unclear whether there is any physiological relevance to GyrB ATPase activation by K^+^ rather than Na^+^.

The observation that eukaryotic topo II enzymes employ the positively charged primary amine group of a Lys side chain in place of a K^+^ ion in the equivalent of site 1 is worthy of note. We sought to investigate the degree of conservation of the key residue, *i.e.* the equivalent of Ser121 in *E. coli* GyrB43 and Lys147 in yeast topo II, and showed that there was very strong conservation of this ‘signature’ amino acid within the respective protein families (§S1.1). Similar substitutions are observed elsewhere in the wider phosphotransferase family. For example, whilst bovine Hsc70 binds K^+^ adjacent to the nucleotide (PDB entry 1hpm; Wilbanks & McKay, 1995 ▶), the structurally related rabbit skeletal muscle actin deploys a Lys side chain in the equivalent position (PDB entry 1atn; Kabsch *et al.*, 1990 ▶). It has previously been suggested in the context of GHKL family enzymes that accounting for monovalent cations in the design of drugs that target the ATPase site could help to minimize undesirable off-target effects (Hu *et al.*, 2003 ▶). Indeed, based on our observations, compounds that overlap both the nucleotide-binding site and site 1 of GyrB could display little or no cross-reactivity with topo II owing to steric clashes with the signature Lys residue.

In summary, we present evidence for two monovalent cation-binding sites in the vicinity of the Mg-ATP site in the ATPase domain of *E. coli* GyrB. One site lies at the dimer interface and in our hands preferentially binds a sodium ion; the functional significance of this site is unclear. The other site shows a preference for binding a potassium ion, which interacts directly with the α-phosphate of the nucleotide, and we postulate that it is responsible for the potassium-stimulated increase in the ATPase activity of DNA gyrase that we observe biochemically.

## 5. Related literature   

The following references are cited in the Supporting Information for this article: Kabsch (1976 ▶); Altschul *et al.* (1990 ▶) and Suzek *et al.* (2007 ▶).

## Supplementary Material

PDB reference: N-terminal 43 kDa fragment of *E. coli* DNA gyrase B, 4wub

PDB reference: 4wuc

PDB reference: 4wud

PDB reference: 4xtj

Supporting Information.. DOI: 10.1107/S1399004715002916/dw5123sup1.pdf

## Figures and Tables

**Table 1:** X-ray data collection and processing Values in parentheses are for the outer shell.

Data set	K-only	Na-only	No-salt	K+Na	K-anom	K+Na-anom
Beamline	I24	I24	I04-1	I04	I24	I24
Wavelength ()	1.033	1.033	0.917	0.979	1.907	1.907
Detector	Pilatus 6M	Pilatus 6M	Pilatus 2M	Q315 CCD	Pilatus 6M	Pilatus 6M
Space group	*C*222_1_	*C*222_1_	*C*222_1_	*C*222_1_	*C*222_1_	*C*222_1_
Unit-cell parameters						
*a* ()	87.88	87.91	87.80	87.52	87.80	87.69,
*b* ()	140.90	140.88	141.48	140.91	142.14	140.93
*c* ()	79.56	79.52	80.30	79.84	79.78	80.00
= = ()	90	90	90	90	90	90
Resolution range ()	36.741.75 (1.801.75)	27.491.90 (1.951.90)	27.451.95 (2.001.95)	35.231.92 (1.971.92)	43.852.90 (2.982.90)	40.002.90 (2.982.90)
Total No. of reflections	298692 (19421)	253416 (17831)	217252 (15998)	199461 (37803)	113599 (7060)	114279 (6936)
Unique reflections	50058 (3685)	39031 (2860)	36686 (2671)	37803 (2668)	11376 (837)	11177 (794)
Completeness (%)	99.9 (99.9)	99.9 (99.8)	99.7 (99.7)	99.6 (96.2)	99.8 (99.6)	98.8 (94.7)
Multiplicity	6.0 (5.3)	6.5 (6.2)	5.9 (6.0)	5.3 (5.3)	10.0 (8.4)	10.2 (8.7)
*I*/(*I*)	19.1 (2.0)	22.4 (2.5)	15.9 (2.3)	18.5 (1.9)	18.1 (3.2)	19.8 (9.3)
*R*_merge_ †	0.048 (0.761)	0.056 (0.986)	0.052 (0.756)	0.054 (0.812)	0.121 (0.722)	0.094 (0.201)
*R*_meas_ ‡	0.059 (0.849)	0.062 (1.114)	0.058 (0.833)	0.061 (0.915)	0.134 (0.804)	0.099 (0.213)
CC_1/2_ §	0.998 (0.727)	0.999 (0.643)	0.999 (0.650)	0.999 (0.699)	0.996 (0.818)	0.995 (0.980)
Wilson *B* value (^2^)	27.9	27.4	32.7	27.5	60.9	42.7

† *R*_merge_ = 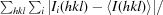 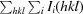.

‡ *R*_meas_ = 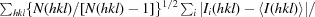 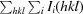, where *I*_i_(*hkl*) is the *i*th observation of reflection *hkl*, *I*(*hkl*) is the weighted average intensity for all observations *i* of reflection *hkl* and *N*(*hkl*) is the number of observations of reflection *hkl*.

§ CC_1/2_ is the correlation coefficient between symmetry-related intensities taken from random halves of the data set.

**Table 2:** Refinement of X-ray structures Values in parentheses are for the outer shell.

Data set	K-only	Na-only	K+Na	No-salt
Resolution range ()	36.741.75 (1.801.75)	27.491.90 (1.951.90)	35.231.92 (1.971.92)	27.541.95 (2.001.95)
Reflections: working/free†	47498/2538	37010/2022	35855/1947	34791/1894
Final *R*_work_ ‡	0.179 (0.292)	0.184 (0.310)	0.184 (0.300)	0.185 (0.396)
Final *R*_free_ §	0.204 (0.337)	0.220 (0.304)	0.218 (0.328)	0.219 (0.410)
Cruickshank DPI	0.095	0.125	0.128	0.130
R.m.s. bond deviations ()	0.009	0.009	0.009	0.010
R.m.s. angle deviations ()	1.25	1.26	1.27	1.34
No. of protein residues [ranges]	384 [4303, 309392]	384 [4303, 309392]	384 [4303, 309392]	384 [4303, 309392]
No. of waters	249	194	220	139
No. of ADPNP molecules	1	1	1	1
No. of Mg^2+^ ions	1	1	1	1
No. of K^+^ ions	1	0	1	0
No. of Na^+^ ions	1	2	1	1
No. of Cl ions	1	1	1	1
Mean *B* factors (^2^)				
Protein	37	37	37	44
Water	41	42	40	45
ADPNP	27	26	28	31
Ions	34	34	34	44
Overall	37	37	37	44
Ramachandran plot§				
Favoured (%)	97.7	97.4	98.2	98.2
Allowed (%)	2.0	2.3	1.5	1.5
Disallowed (%)	0.3	0.3	0.3	0.3
PDB code	4wub	4wuc	4xtj	4wud

† The data set was split into ‘working’ and ‘free’ sets consisting of 95 and 5% of the data, respectively. The free set was not used for refinement.

‡ The *R* factors *R*_work_ and *R*_free_ are calculated as follows: *R* = 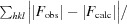 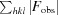 100, where *F*_obs_ and *F*_calc_ are the observed and calculated structure-factor amplitudes, respectively.

§ As calculated using *MolProbity* (Chen *et al.*, 2010 ▶).

**Table 3:** Summary of selected homologous PDB entries described in this study

Protein	Source	PDB code	Resolution ()	Aligned residues†	R.m.s. deviation† ()	Identity† (%)	Site 1 occupancy	Reference
GyrB43	*E. coli*	4pu9	2.40	353	0.69	99.7	Water	Stanger *et al.* (2014 ▶)
GyrB43	*E. coli*	1ei1	2.30	383	0.52	99.5	Water	Brino *et al.* (2000 ▶)
ParE	*E. coli*	1s16	2.10	354	1.37	36.2	Mg^2+^ ‡	Bellon *et al.* (2004 ▶)
Topo II	*S. cerevisiae*	1pvg	1.80	302	2.04	24.8	N of Lys	Classen *et al.* (2003 ▶)
Topo II	*H. sapiens*	1zxn	2.51	281	1.97	22.1	N of Lys	Wei *et al.* (2005 ▶)
MutL	*E. coli*	1nhi	2.00	270	3.18	15.9	K^+^	Hu *et al.* (2003 ▶)
Topo VIB	*S. shibatae*	1mx0	2.30	235	2.28	15.7	N of Lys	Corbett Berger (2003 ▶)
BCK	*R. norvegicus*	1gkz	2.20	107	1.95	21.5	K^+^	Machius *et al.* (2001 ▶)

† Values determined using the *Secondary Structure Matching* (*SSM*) algorithm within *Coot* (Emsley Cowtan, 2004 ▶) to superpose structures on the K-only GyrB43 model.

‡ Could be modelled as K^+^.

**Table 4:** Site 1 occupancies and cavity dimensions

		Dimensions† ()				
Model	Occupancy	ADPNPPOVal97O	Ile94OGly117O	Ser121OAla100O	Average OO distance ()	Approximate cavity radius[Table-fn tfn10] ()
K-only	K^+^	5.64	5.38	5.65	5.56	1.38
Na-only	Na^+^	6.21	5.63	6.01	5.95	1.58
K+Na	K^+^	5.65	5.44	5.65	5.58	1.39
No-salt	Water	6.03	5.61	6.02	5.89	1.54

† See Supplementary Fig. S3(*a*).

‡ See Supplementary Fig. S3(*b*).
