# Supplementary material for: The role of monovalent cations in the ATPase reaction of DNA gyrase
Source: Acta Crystallogr D Biol Crystallogr. 2015 Mar 27;71(Pt 4):996–1005. doi: 10.1107/S1399004715002916 (PMC4388272; doi:10.1107/S1399004715002916)
Supplement: Supplementary file 1 [file d-71-00996-sup1.pdf]

BIOLOGICAL CRYSTALLOGRAPHY

Volume 71 (2015)

Supporting information for article:

The role of monovalent cations in the ATPase reaction of DNA gyrase

Stephen James Hearnshaw, Terence Tsz-Hong Chung, Clare Elizabeth Mary Stevenson, Anthony Maxwell and David Mark Lawson

S1. Supplementary methods

S1.1. Conservation of the GHKL site 1 "signature" residue

The conservation of the site 1 "signature" residue was investigated within the GyrB and topo II families through the alignment of hits obtained from *BLAST* (http://web.expasy.org/blast/)(Altschul et al., 1990) searches of the UNIREF50 UniProt non-redundant reference protein sequence database (http://www.embnet.sk:8080/srs81//srs?page=libinfo&libName=UNIREF50)(Suzek et al., 2007). The query sequences were *E. coli* GyrB (UniProtKB/Swiss-Prot entry P0AES6) and yeast DNA topoisomerase II (UniProtKB/Swiss-Prot entry P06786), and the expectation value (E) threshold was set at the default value of 10. For GyrB, 93 sequences were returned that included the region containing the signature residue, and the most distant sequence shared 39% identity with the query. Within these sequences, 85 had Ser as the signature residue and, in six, it was semi-conservatively substituted to Thr. However, for two sequences, the residue was Lys (UniProtKB/Swiss-Prot entries B2URM5 and W0JA05), but they were still annotated as GyrB subunits and had a substantially greater identity to the query GyrB sequence (41% and 48%, respectively) than to the query topo II sequence (16% and 15%, respectively). For the topo II *BLAST* search, 98 sequences were returned with the most distant being 37% identical to the query; this time the signature Lys residue was universally conserved.

Table S1 Theoretical anomalous scattering factors calculated by the Cromer and Liberman's method for selected common elements at a wavelength of 1.91 Å

Element	Anomalous signal (electrons) †
K	1.55
Cl	1.03
S	0.83
P	0.65
Mg	0.27
Na	0.19
O	0.05
N	0.03
C	0.01

† Data taken from http://lipro.msl.titech.ac.jp/scatfac/scatfac.html and listed in order of decreasing anomalous signal.

Table S2 Pairwise comparisons of GyrB43 structures presented in this study

R.m.s. deviation (Å) †	Na-only	K+Na	No Salt
K-only	0.156 (0.183)	0.129 (0.061)	0.242 (0.148)
Na-only	-	0.171 (0.171)	0.225 (0.084)
K+Na	-	-	0.188 (0.132)

† Values calculated after superposition using *LSQKAB* (Kabsch, 1976).

‡ Values in parentheses are for the ATP-lids only (residues 90-126 inclusive).

Table S3 Statistics for Site 1 and Site 2 in K-only model

Site 1: Occupancy = K^+; B-factor = 27.1 Å^2			Site 2: Occupancy = Na^+; B-factor = 32.1 Å^2		
Liganding atom	B-value (Å^2)	Interaction length (Å)	Liganding atom	B-value (Å^2)	Interaction length (Å)
Ile94 O	26.3	2.69	Lys103 O	31.0	2.80
Val97 O	28.3	2.95	Asp105 O	36.9	2.73
Ala100 O	26.9	2.93	Water O	33.5	2.29
Gly117 O	23.4	2.70	Water O	33.3	2.39
Ser121 Oγ	26.9	2.80	Water O	35.5	2.32
ADPNP PαO	24.1	2.78	Water O	45.1	2.44
Averages	26.0	2.81	Averages	35.9	2.50
Ligand B/ average B (%)	104	-	Ligand B/ average B (%)	89	-

Table S4 Statistics for Site 1 and Site 2 in Na-only model

Site 1: Occupancy = Na^+; B-factor[‡] = 27.8/24.1 Å^2			Site 2: Occupancy = Na^+; B-factor = 40.3 Å^2		
Liganding atom	B-value (Å^2)	Interaction length[†] (Å)	Liganding atom	B-value (Å^2)	Interaction length (Å)
Ile94 O	30.7	2.54	Lys103 O	31.9	2.64
Val97 O	29.8	2.28	Asp105 O	38.1	2.81
Ala100 O	28.0	3.04	Water O	39.6	2.24
Gly117 O	25.4	3.13	Water O	41.9	2.44
Ser121 Oγ	32.6	2.35	Water O	36.4	2.25
ADPNP PαO	23.6	2.65	Water O	39.8	2.40
Averages	28.4	2.67	Averages	38.0	2.46
Ligand B/ average B (%)	98/85[‡]	-	Ligand B/ average B (%)	106	-

[†] Only values to the closest Na^+ position are shown.

[‡] Values for each half-occupancy site.

Table S5 Statistics for Site 1 and Site 2 in K+Na model

Site 1: Occupancy = K^+; B-factor = 29.2 Å^2			Site 2: Occupancy = Na^+; B-factor = 33.6 Å^2		
Liganding atom	B-value (Å^2)	Interaction length (Å)	Liganding atom	B-value (Å^2)	Interaction length (Å)
Ile94 O	25.0	2.72	Lys103 O	31.7	2.62
Val97 O	30.0	2.89	Asp105 O	35.5	2.79
Ala100 O	27.8	2.95	Water O	32.1	2.38
Gly117 O	24.4	2.72	Water O	31.6	2.38
Ser121 Oγ	28.8	2.78	Water O	32.2	2.36
ADPNP PαO	26.3	2.83	Water O	43.3	2.41
Averages	27.1	2.82	Averages	34.4	2.49
Ligand B/ average B (%)	108	-	Ligand B/ average B (%)	98	-

Table S6 Statistics for Site 1 and Site 2 in No-salt model

Site 1: Occupancy = Water; B-factor = 36.9 Å^2			Site 2: Occupancy = Na^+; B-factor = 44.3 Å^2		
Liganding atom	B-value (Å^2)	Interaction length (Å)	Liganding atom	B-value (Å^2)	Interaction length (Å)
Ile94 O	36.2	2.75	Lys103 O	36.8	2.56
Val97 O	36.2	(3.38)	Asp105 O	41.4	2.65
Ala100 O	32.4	3.05	Water O	40.1	2.29
Gly117 O	28.7	2.97	Water O	48.4	2.44
Ser121 Oγ	34.7	3.22	Water O	40.0	2.40
ADPNP PαO	28.4	2.83	Water O	47.9	2.48
Averages	32.8	2.96[†]	Averages	42.4	2.47
Ligand B/ average B (%)	113	-	Ligand B/ average B (%)	104	-

[†] Average calculated by excluding value in brackets.

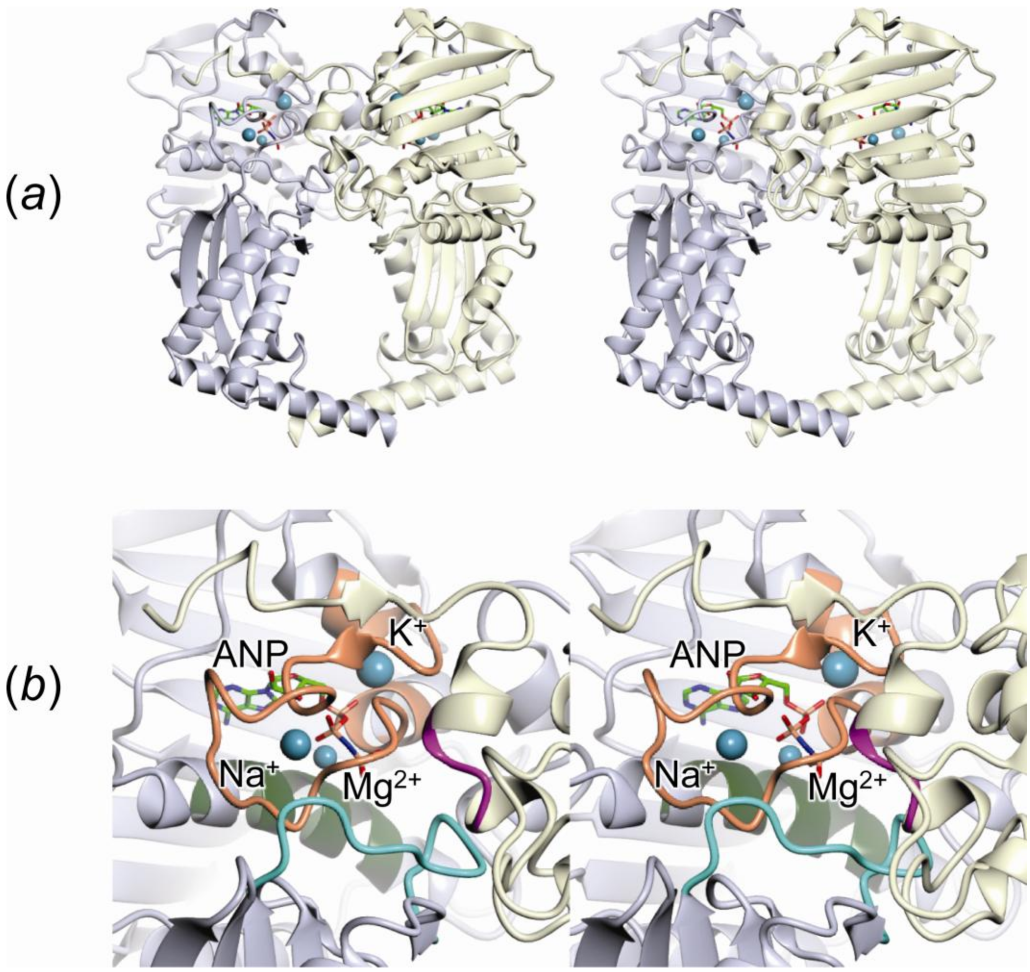

Figure S1 Stereoviews giving an overview of cation binding sites in *E. coli* GyrB43. (*a*) Cartoon of the K-only model of the GyrB43 homodimer. (*b*) Enlarged view of the top left hand corner of (*a*) showing the relative positions of the metal binding sites and the bound ADPNP (abbreviated to ANP). The slate grey and pale yellow colours define the two subunits, whilst the additional colours indicate the regions that participate in cation binding, as detailed in Fig. 1*b*.

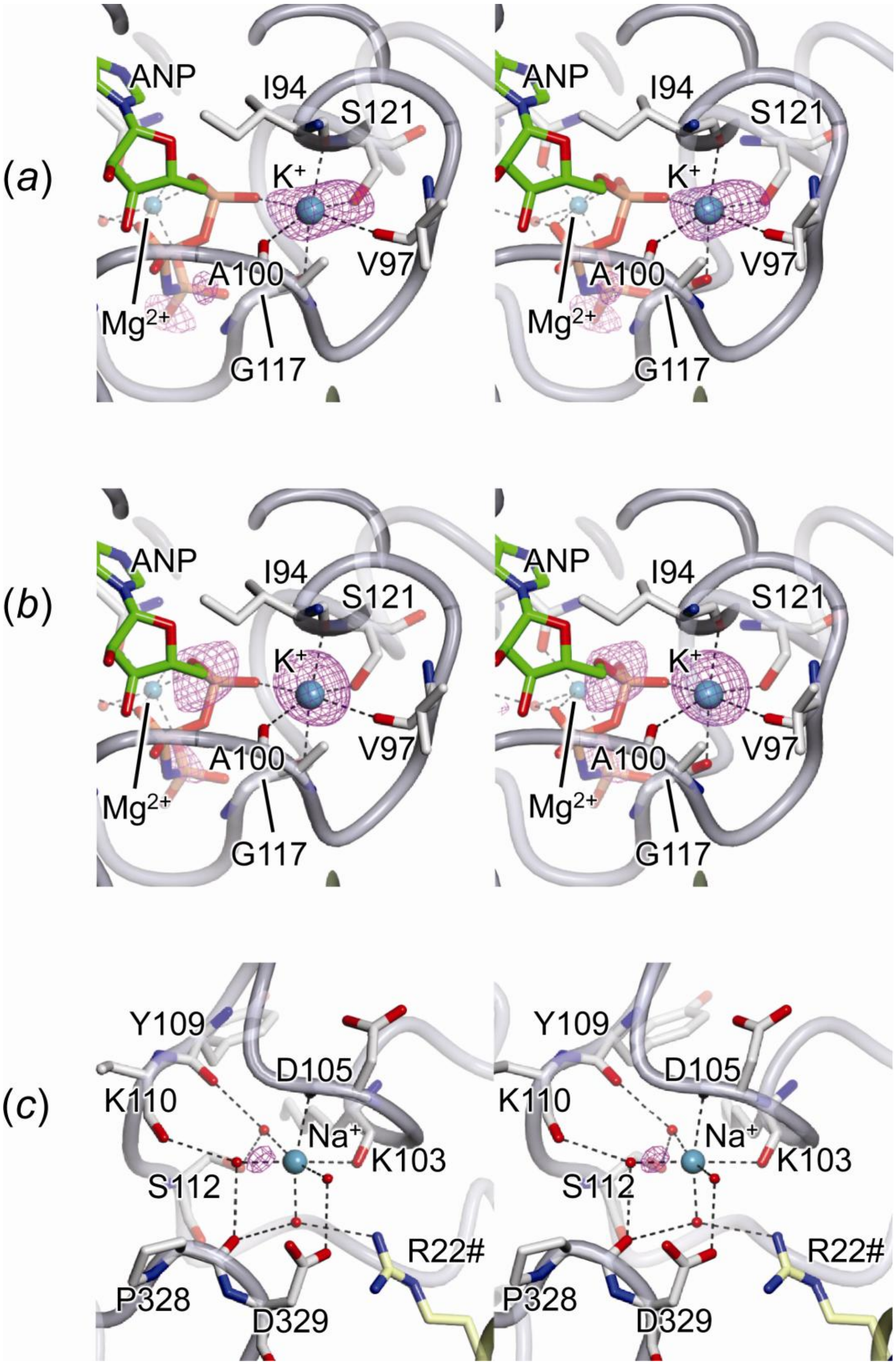

Figure S2 Stereoviews showing anomalous difference Fourier peaks in and around the cation binding sites in *E. coli* GyrB43 and superposed on the K-only model. These were calculated as described in Materials and Methods and contoured at 4.0 σ. (*a*) Peaks at site 1 derived from the K-anom dataset. (*b*) Peaks at site 1 derived from the K+Na-anom dataset. (*c*) Peaks at site 2 derived

from the K-anom dataset. No peaks were observed in the vicinity of this site in the anomalous difference Fourier calculated from the K+Na-anom dataset when contoured at 4.0 σ (not shown).

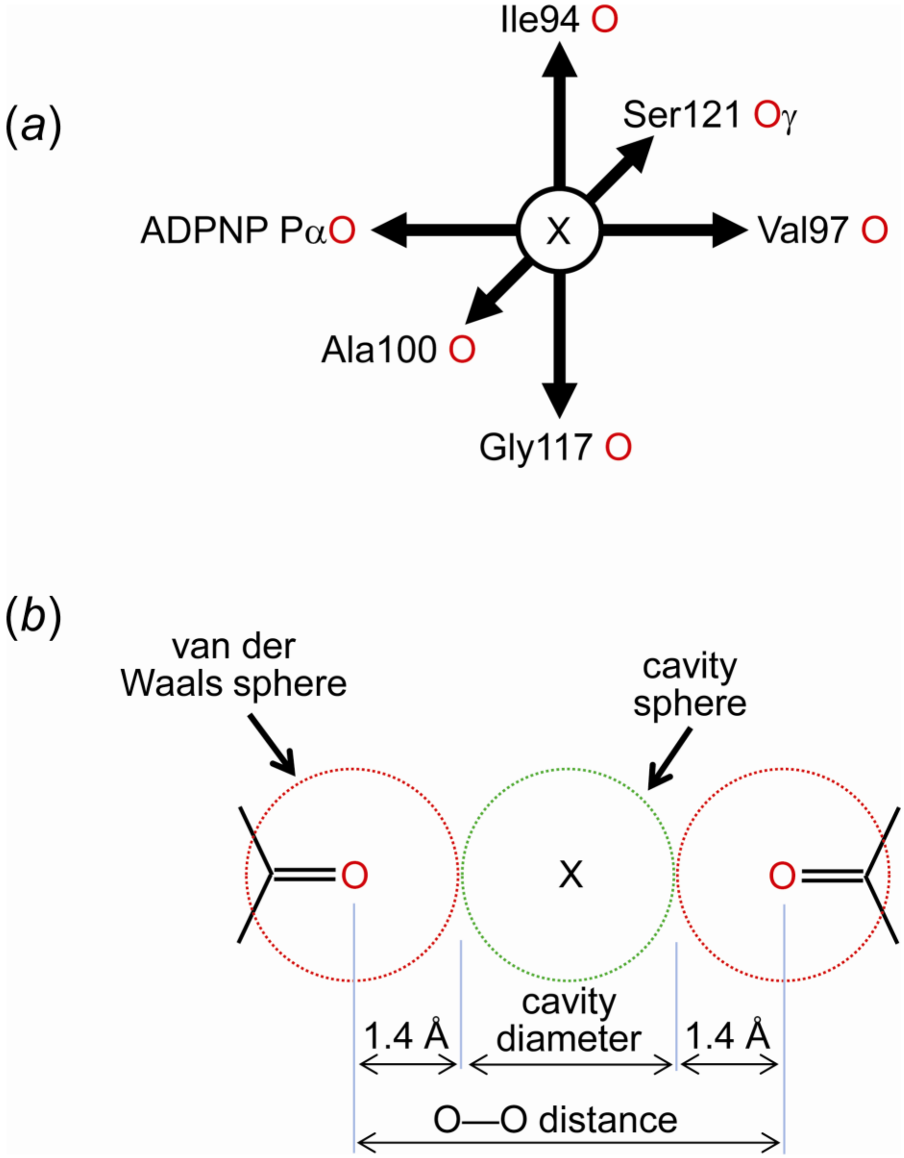

Figure S3 Defining the coordination sphere at site 1 of *E. coli* GyrB43. (a) The six coordinating oxygens adopt an octahedral arrangement around the ligand (denoted “X”). (b) Cavity radii for the four GyrB43 models were estimated by averaging the O—O distances of diametrically opposed liganding atoms, subtracting two times the van der Waals radius for oxygen (i.e. 2 x 1.4 Å), then halving the result. The data are presented in Table 4.
